# *EpiTEome*: Simultaneous detection of transposable element insertion sites and their DNA methylation levels

**DOI:** 10.1186/s13059-017-1232-0

**Published:** 2017-05-12

**Authors:** Josquin Daron, R. Keith Slotkin

**Affiliations:** Department of Molecular Genetics, The Ohio State University, Columbus, OH USA

**Keywords:** Methylome, Transposable elements, Insertion site, Bisulfite, Split reads, Bioinformatics, MethylC-seq

## Abstract

The genome-wide investigation of DNA methylation levels has been limited to reference transposable element positions. The methylation analysis of non-reference and mobile transposable elements has only recently been performed, but required both genome resequencing and MethylC-seq datasets. We have created *epiTEome*, a program that detects both new transposable element insertion sites and their methylation states from a single MethylC-seq dataset. *EpiTEome* outperforms other split-read insertion site detection programs, even while functioning on bisulfite-converted reads. *EpiTEome* characterizes the previously discarded fraction of DNA methylation at sites of new insertions, enabling future investigation into the epigenetic regulation of non-reference and transposed elements.

## Background

Transposable elements (TEs) are mobile fragments of DNA that inhabit the nuclear genome of all eukaryotes. TEs are dynamic, insert into chromosomal loci, generate polymorphisms, and create mutations in protein-coding genes [1, 2]. TE sequences have diversified and are classified by families with common sequences, domains, structures, and mobilization strategies [3, 4]. Some TE families have been evolutionarily successful, going through bursts of activity [5], which result in the expansion and rearrangement of genomes [6, 7]. Due to their ability to create rearrangements and mutations, TEs are targeted for epigenetic silencing. Multiple overlapping mechanisms recognize TE sequences and modify their chromatin with DNA and histone methylation, resulting in the formation of heterochromatin that lacks protein-coding expression (reviewed in [8]).

5-Methylcytosine is a DNA modification targeted to TEs to inhibit their transcriptional activity. DNA methylation is originally targeted to cytosines (Cs) in any sequence context by the activity of small RNA-directed DNA methylation (RdDM) (reviewed in [8]). Once established in plants and vertebrates, DNA methylation can be copied and epigenetically maintained at the TE by the activity of methyltransferase proteins. In plants, DNA methylation is maintained at different levels by distinct methyltransferase proteins in the CG, CHG, and CHH sequence contexts (where H = A, C, or T). DNA methylation is detected by bisulfite sequencing, where non-methylated cytosines are chemically converted and appear as thymine via DNA sequencing, while methylated cytosines are not converted. Bisulfite-conversion whole-genome sequencing (MethylC-seq) is performed by subjecting an adapter-ligated genomic library to bisulfite conversion before library amplification and sequencing [9]. The standard method of MethylC-seq data analysis involves mapping MethylC-seq reads to a reference genome. Consequently, DNA methylation levels of non-reference and mobile TE positions are overlooked.

The detection of non-reference and mobile TE insertion sites has been traditionally performed individually on single TE families [10]. However, whole-genome sequencing has enabled TE insertion site detection of all TE families simultaneously. The preferred approach would be to sequence various genomes and *de novo * assemble each one, identifying polymorphic TE insertion sites. However, short read-length, genome complexity, and high cost prohibit this strategy. Alternatively, genome resequencing and alignment to an available assembled reference genome sequence has been used to detect TE insertion sites. This is accomplished by focusing on the sequencing reads that do not match the reference genome using a split-read mapping strategy to align one end of a single read to the reference genome, while the other half of the read maps to a known TE end [11]. Two such programs are SPLITREADER and TEPID, which have successfully detected TE insertion sites across the resequencing of 216 Arabidopsis natural ecotypes, identifying evolutionarily active TE copies and transposition hotspots [12, 13]. These new insertions sites are generally excluded from the genome-wide analysis of DNA methylation by MethylC-seq. Only recently has the genome-wide DNA methylation of new TE insertion sites been assayed; however, this required both whole-genome resequencing and MethylC-seq datasets [12].

We aimed to utilize the vast and available MethylC-seq data the epigenomics community generates to identify new TE insertion sites rather than resequencing these genomes. We have combined the fields of DNA methylation and TE insertion site detection by creating a program called *epiTEome*. This program can for the first time identify a TE insertion from MethylC-seq reads, as well as determine the methylation state of the new TE insertion and surrounding insertion site. This program circumvents the necessity to perform genome resequencing to identify new TE insertion sites, reducing the required cost to analyze TE insertion site and DNA methylation data to a single MethylC-seq experiment.

## Results

### Description of the *epiTEome* program

**Fig. 1:**
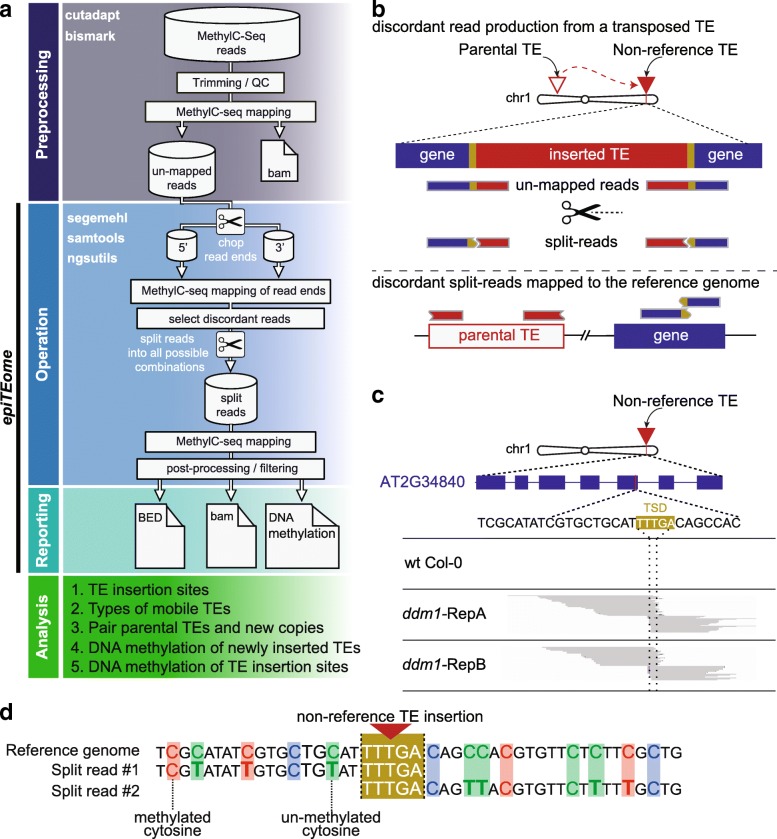
Design of *epiTEome* function. **a** *Workflow* of methodology developed to identify non-reference insertions of TEs using filtered MethylC-seq reads that fail to align to the reference genome. **b** Principle behind split-read detection of new TE insertion sites. Reads that fail to fully map to the reference genome are used to identify the sites of new TE insertion. Non-mapping reads are split and mapped to the reference genome to identify reads with one end that maps to a TE and the other end to the site of insertion. **c** Example of a new TE insertion detected by *epiTEome* in Arabidopsis: *ddm1* mutants undergo TE transcriptional reactivation and transposition [30]. Split reads not present in wild-type (wt Col-0) identify a TE insertion into the gene At2g34840 in two biological replicates of *ddm1* MethylC-seq (RepA and RepB). The 5′ and 3′ flanking spit reads overlap (*dashed lines*) at the target site duplication (*gold sequence*) generated by TE insertion. **d** In addition to identifying new TE insertion sites, *epiTEome* detects the cytosine methylation status at these loci. Sequence alignment of split MethylC-seq reads at the insertion site are used to determine the cytosine DNA methylation status. Unconverted cytosines represent methylated bases, while C → T transitions (*bold*) in the MethylC-seq reads represent unmethylated cytosines. The sequence context of each cytosine is displayed (CG = *red*, CHG = *blue*, CHH = *green*)

Unlike other programs developed to identify new TE insertion sites, *epiTEome* was developed to initiate analysis with MethylC-seq reads generated from whole-genome sequencing of bisulfite-converted DNA. Before mapping, reads are trimmed and processed to remove adapters, low quality and imperfect sequencing reads from a FASTQ file (preprocessing, Fig. 1a). The trimmed and filtered reads are then mapped to the reference genome using *Bismark* [14] or any MethylC-seq mapping program. Stringent filtering and sensitive mapping are suggested to reduce the fraction of low quality unmapped reads, as the MethylC-seq reads that fail to map to the reference genome are the input to *epiTEome* (Fig. 1a).

*EpiTEome* splits and maps each MethylC-seq read that failed to align to the reference genome. The initial length of each spit-read end is user-defined; however, it should be over 25 nucleotides (nt). *EpiTEome* first identifies the reads with discordant (map to different locations in the genome) ends using the mapping program *Segemehl* [15] (operation, Fig. 1a). Once the discordant reads are identified, the corresponding full-length read is split into all possible combinations with a minimal length of 25 nt. Each variation of the split-read is mapped to the reference genome to identify the breakpoint location on the read where one half maps to a TE and the other half to the new insertion site (Fig. 1b). This process identifies the point of the read that transitions from one discordant position to another and only the read split at this position is retained for analysis of TE insertion sites (Fig. 1c) and DNA methylation (Fig. 1d).

Discordant split reads are processed by filtering for those with at least one end at the edge of an annotated TE (operation, Fig. 1a). If both ends discordantly map to the same TE family (likely due to frequent TE internal deletions), the read is discarded. Discordant reads are next clustered based on their location in the genome and further filtered. Read clusters are filtered for: (1) the number of split-reads supporting the new insertion site (>5); (2) both ends of the same TE must be represented at the insertion site; and (3) the overlap of the reads at the insertion site should not extend beyond the target site duplication (TSD) generated by TE insertion (Fig. 1c). *EpiTEome* results are reported as coordinate positions of each TE insertion site, TE family, and parental TE copy (reporting, Fig. 1a). Application of this workflow identifies sites of new TE insertion, the TE TSD, and mobile TE families (analysis, Fig. 1a).

To this point in the workflow, *epiTEome* functions similarly to SPLITREADER and TEPID to identify new TE insertion sites (Fig. 1b). The added value of *epiTEome* is the ability to detect the DNA methylation status of the transposed TE and insertion site using the exact split-reads that identified the transposition event. DNA methylation is reported as single-insertion alignments (Fig. 1d), as well as meta-analysis of all insertion sites in the sample. The methylation is split into CG, CHG, and CHH contexts for both the 5′ and 3′ (non-TE) flank of the insertion site (Fig. 1d), as well as the 5′ and 3′ TE ends (analysis, Fig. 1a).

### Detection of simulated TE insertions

**Fig. 2:**
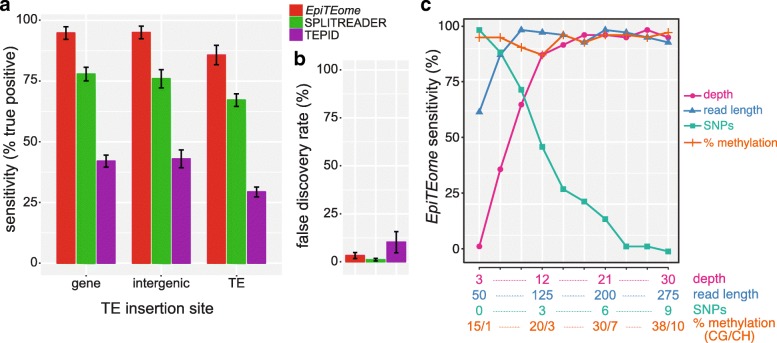
Validation of *epiTEome* on simulated data. **a** *Bar plot* of sensitivity of detection for simulated TE insertions at three different TE insertion contexts (gene, intergenic, TE). SPLITREADER and TEPID use non-bisulfite converted reads, while *epiTEome* utilizes bisulfite-converted MethylC-seq reads. **b** FDR of *epiTEome*, SPLITREADER, and TEPID calculated from the same simulated data as part A. *Error bars* in (**a**) and (**b**) represent the 95% confidence interval (CI) generated using five replicates. **c** Analysis of how the variables of sequencing depth, read length, methylation level, and number of SNPs affect *epiTEome* sensitivity. Throughout the analysis in (**c**), *epiTEome* produced a 2.88% false-positive average, with a standard deviation of 1.45

To test the sensitivity (true positive [TP] rate) of *epiTEome*, we created 84 synthetic (simulated) TE insertions into Arabidopsis chromosome 2. We chose a plant genome because plants methylate cytosines in all sequence contexts (CG, CHG, CHH), producing a computational challenge in MethylC-seq analysis. Synthetic insertions represented the seven types of mobile TE families previously identified in genome-wide transposition experiments across the *Arabidopsis thaliana* species range: Copia elements (ATCOPIA78/*Onsen* and ATCOPIA93/*Evadé*), Gypsy elements (ATGP2 and ATHILA2), AtEnSPM elements (ATENSPM5 and ATENSPM6), Mutator elements (VANDAL6 and BOMZH1), Helitron elements (ATREP2A and ATREP11A), hAT elements (SIMPLEHAT2 and ATHATN2), and the LINE element (ATLINE2) [12, 13]. We created simulated insertions into the chromosomal contexts of genes, TEs, and non-TE/non-gene “intergenic” regions. We tested SPLITREADER and TEPID using *in silico* generated DNA sequencing reads and *epiTEome* on the same dataset that we *in silico* bisulfite-converted assuming the genome-wide rates of CG and CH methylation (see “Methods”). We found that TE insertions were detected at 92% sensitivity by *epiTEome* using bisulfite-converted reads, a higher sensitivity compared with programs that do not use bisulfite-converted reads (Fig. 2a). For insertions into repetitive regions, reduced sensitivity occurs for each of the programs tested, including a drop from 95% (insertion into a gene) to 86% (insertion into a TE) sensitivity for *epiTEome* (Fig. 2a). However, *epiTEome* remains the most sensitive TE insertion site detection program independent of TE insertion site (Fig. 2a), even while using bisulfite-converted MethylC-seq reads. We also calculated the false discovery rate (FDR) of *epiTEome* (3.26%) and demonstrate that it is comparable to SPLITREADER and TEPID (Fig. 2b). We therefore conclude that *epiTEome* is a comparably sensitive and accurate detector of TE insertion sites, while using the distinct MethylC-seq data source.

To determine the factors responsible for accurate TE insertion detection from MethylC-seq data by *epiTEome*, we tested *epiTEome* sensitivity while altering four parameters: sequencing depth; read length; methylation level; and number of non-bisulfite conversion-induced single nucleotide polymorphisms (SNPs). We *in silico* distributed 84 TE copies randomly on Arabidopsis chromosome 2 and independently tested each of the four variables (see “Methods”). We found that the level of methylation and read length are not critical, as long as the read length is over 75 nt (post-trimming) (Fig. 2c). More critical are the depth of sequencing/genome coverage and the number of (non-bisulfite-induced) SNPs present between the reads and reference sequence. Therefore, as a best practice to identify TE insertions from MethylC-seq data, we suggest sequencing using ≥ 100 nt length reads, depth of ≥ 18x, while using a closely related reference genome.

### *EpiTEome *accurately detects TE insertions from MethylC-seq data

**Fig. 3:**
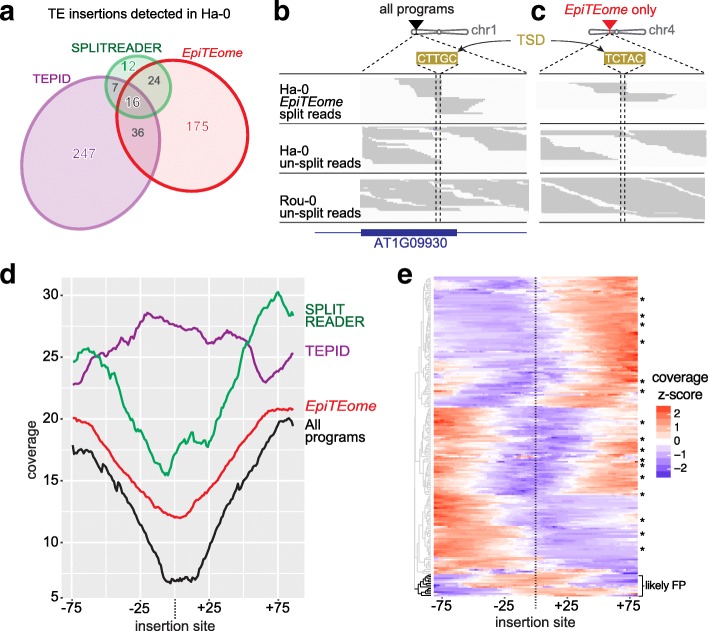
Validation of *epiTEome* using published MethylC-seq data. **a** *Venn diagram* comparing three independent programs created to identify TE insertion sites in the Arabidopsis ecotype Ha-0. *EpiTEome* is the only program that utilizes MethylC-seq data. Color codes are maintained throughout panels (**b**)–(**d**). Split-reads identify the insertion and target site duplication of a TE insertion detected by all three programs (**b**) and a TE insertion specifically detected by *epiTEome* (**c**). The split-read analysis is confirmed by the decrease in coverage of un-split MethylC-seq reads in Ha-0 (insertion present) vs. the ecotype Rou-0 (insertion absent). **d** *Meta-plot* of MethylC-seq un-split read coverage at the TE insertion sites and flanking regions uniquely detected by each program or detected by all three. **e** MethylC-seq un-split read coverage z-score for each of the 175 TE insertions uniquely identified by *epiTEome*, plus the 16 detected by all three programs (*asterisks*). Seven percent of the insertion sites with high un-split read coverage (*bracket*) at the TE insertion site are likely false positives (FP)

To determine if *epiTEome* can detect TE insertions from biologically relevant MethylC-seq data, we investigated naturally occurring Arabidopsis ecotypes because these samples have been subjected to both genome resequencing and MethylC-seq analyses [16–18]. We chose the Ha-0 and Rou-0 closely related ecotypes because both SPLITREADER and TEPID have previously identified unique insertions in Ha-0 compared with Rou-0 and the reference Col-0 ecotype based on genome resequencing data [12, 13]. *EpiTEome* was launched on publicly available MethylC-seq reads from Ha-0 (85 nt reads, 21x coverage, 0.31 SNPs/read compared to the Col-0 reference calculated in [18]) [17]. The overlap between identified Ha-0 TE insertions between all three programs is shown in Fig. 3a. Sixteen TE insertions were identified by all programs (black), including the ATCOPIA93 (*Evadé*) insertion into At1g09930 (creating the target site duplication CTTGC) shown in Fig. 3b. Therefore, *epiTEome* can successfully detect known TE insertion sites from the unique MethylC-seq data source.

Although *epiTEome*, SPLITREADER, and TEPID identified some non-reference TE insertion sites in common, many insertions were uniquely identified by only one program (Fig. 3a). To determine if a detected TE insertion site is real, we performed a verification analysis based on the mapping coverage of the un-split reads. For example, due to the TE insertion into At1g09930 in the Ha-0 ecotype, un-split read coverage at this site is absent compared with the flanking DNA. This lack of un-split read coverage is due to all of the reads from this region having TE content and thus not mapping the reference genome that lacks the TE insertion (Fig. 3b). This is in contrast to the full un-split read coverage of the closely related ecotype Rou-0 that does not carry the TE insertion (Fig. 3b). TE insertions detected by split-reads can therefore be confirmed by a reduced coverage of un-split reads at the insertion site compared to the flanking DNA.

We next investigated a TE insertion site only detected by *epiTEome*. An ATCOPIA58 insertion into chromosome 4 was detected in the MethylC-seq split reads by *epiTEome* and is verified by the lack of un-split read coverage at the insertion site (compared to Rou-0 which does not have the TE insertion) (Fig. 3c). To determine if a majority of the 175 TE insertions identified only by *epiTEome* (Fig. 3a) are true positives, we performed a meta-analysis of the un-split MethylC-seq read coverage of TE insertion sites that were detected by all programs (black), or uniquely by *epiTEome* (red), TEPID (purple) or SPLITREADER (green) (Fig. 3d). We find the highest confidence in the TE insertions detected by all three programs, while the unique TE insertions detected by *epiTEome* (from MethylC-seq) are supported by decreased un-split read coverage at the insertion site. The 12 TE insertion sites uniquely detected by SPLITREADER are likely missed by *epiTEome* (false negatives [FNs]), while the 247 insertions uniquely identified by TEPID are not supported in the MethylC-seq data. These unconfirmed insertions detected by TEPID are likely not due to the accuracy of the program, but rather due to the fact that TEPID TE insertions are identified based on genome resequencing, not the MethylC-seq dataset that we are testing it against. To further determine the rate at which *epiTEome* was detecting FPs, we investigated the un-split MethylC-seq read coverage of each of the TE insertion sites uniquely detected by *epiTEome* or detected by all three programs. This analysis demonstrates that a decrease in un-split read coverage occurs for 93% of these TE insertion sites (Fig. 3e), providing confidence that the majority of TE insertions detected by only *epiTEome* are true positives from the MethylC-seq raw data.

### Detection of new TE insertions in repetitive crop genomes

**Fig. 4:**
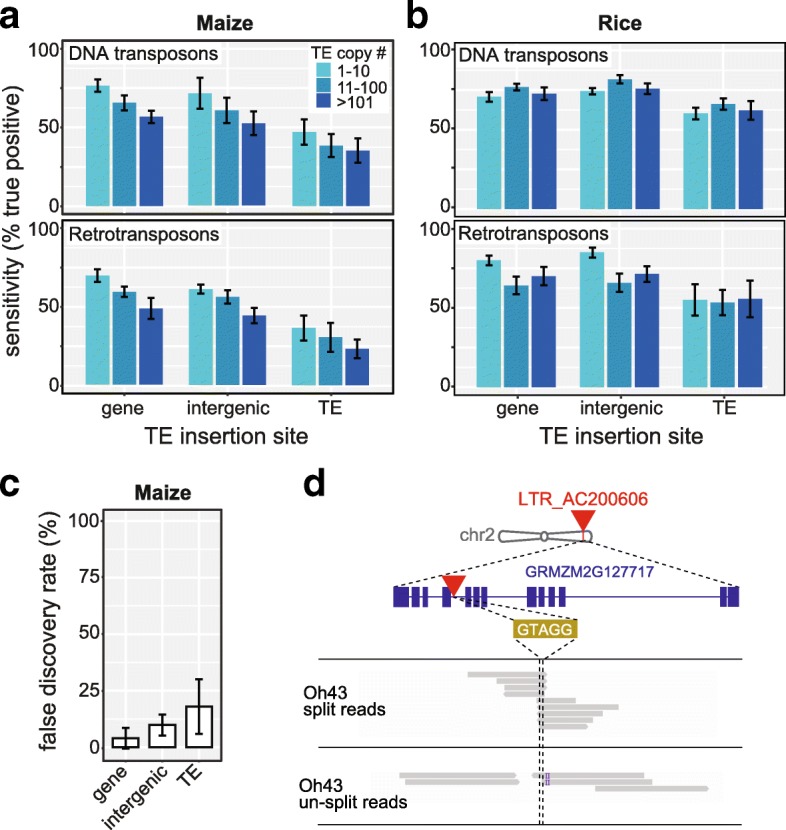
*EpiTEome* detects new TE insertions in repetitive crop genomes. **a**, **b** *Bar plot* of sensitivity of detection for simulated TE insertions at three different potential TE insertion contexts (gene, intergenic, TE) in the maize (**a**) or rice (**b**) genomes using *in silico* generated bisulfite-converted reads. Results are divided by TE copy number. **c** FDR of *epiTEome* calculated from the same simulated data as part A. *Error bars* in (**a**)–(**c**) represent the 95% CI generated using five replicates. **d** Genome browser visualization of a non-reference (not in the reference B73 genome) LTR retrotransposon TE insertion into the PFK5 GRMZM2G127717 gene in the Oh43 inbred line identified by *epiTEome*

To determine if *epiTEome* could function to identify TE insertions within larger and more complex plant crop genomes, we performed a simulated detection of new TE insertions in maize (2.3 Gbp, 85% TEs [19]) and rice (389 Mbp, ~35% TEs [20]). As expected, *epiTEome* functions with the highest sensitivity on low copy number TEs inserted into genic regions (Fig. 4a, b). When the retrotransposon copy number exceeds 100, and when inserted into TE regions of the genome, the sensitivity of *epiTEome* drops to 25% in maize and 56% in rice (Fig. 4a, b). This sensitivity drop represents the computationally most difficult TE insertions to detect and these TE-into-TE insertion events are associated with an increase in FDR (Fig. 4c). This suggests that *epiTEome* will function better for less repetitive genomes or when performed on highly repetitive genomes such as maize *epiTEome* will be most sensitive to detect insertion of DNA TEs into genes.

To test *epiTEome* on biological data, we investigated published maize MethylC-seq data to identify TE insertion variation in the standard inbred line Oh43 compared with the reference genome strain B73. We chose Oh43 because it represents a computational challenge due to the large variation (non-bisulfite-induced SNPs) between maize inbred lines. In addition, the Oh43 MethylC-seq had not been subjected to target enrichment via sequence capture, but rather represented the whole genome at 13x coverage [21]. We found 18 TE insertions (eight into genes, Table 1) in Oh43 compared with B73. For example, we identified an LTR retrotransposon TE inserted into an intron of the PFK5 protein-coding gene, which was verified as a gap in un-split read coverage at this site (Fig. 4d). This analysis demonstrates that *epiTEome* is capable of detecting TE insertions from MethylC-seq datasets produced from repetitive crop genomes.

**Table 1:** *EpiTEome*-identified non-reference TE insertions in maize Oh43 genes compared to the B73 reference

Gene ID	Gene annotation	Chromosome	Insertion site	Genic location	TE family	TE superfamily	Supporting spit reads
GRMZM2G127717	Phosphofructokinase 5 (PFK5)	2	181762712	Intron	LTR_AC200606	Unknown LTR	9
GRMZM2G343360	SAUR-like auxin-responsive protein family	2	197668412	Exon	LTR_AC191075	Unknown LTR	5
GRMZM2G014994	GPI-anchored protein	4	163300669	Intron	Gypsy-173_ZM-LTR	Gypsy LTR	7
GRMZM2G178753	Kinase-like protein	5	19547332	Exon	Gypsy-143_ZM-LTR	Gypsy LTR	5
GRMZM2G330684	Ring Zinc Finger	5	30857925	Intron	HARB-N3_ZM	Harbinger	5
GRMZM2G148229	SNARE associated Golgi protein family	7	170575334	Intron	GYZMA1_LTR	Gypsy LTR	5
GRMZM5G871262	LIMR family protein	9	24960353	Intron	Gypsy29-ZM_LTR	Gypsy LTR	7
GRMZM2G111066	Protein SIP5 isoform X1	9	105921345	exon	Gypsy29-ZM_LTR	Gypsy LTR	6

### *EpiTEome* identifies the cytosine methylation status of non-reference TEs and flanking DNA

**Fig. 5:**
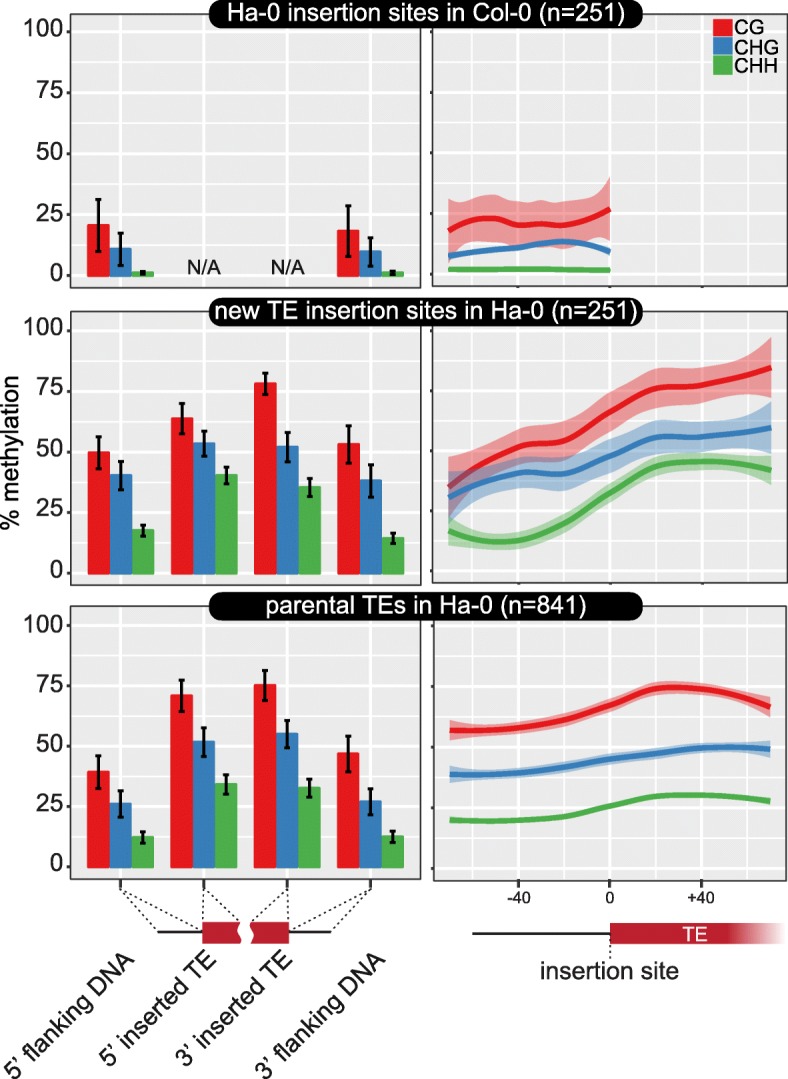
*EpiTEome* detects DNA methylation at new TE insertion sites. Average DNA methylation at new insertions in the Arabidopsis Ha-0 ecotype (*middle*), these same sites without TE insertion in the reference Col-0 ecotype (*top*), and the parental TEs in Ha-0 that produced each transposed TE (*bottom*). DNA methylation is split between cytosine sequence contexts (*colors*) and location at the insertion site (*x-axis*). *Bar plots* are shown on the *left* and a *metaplot* combining both the 5′ and 3′ TE ends and insertion sites is shown on the *right. Error bars* (*left*) and transparent colors (*right*) represent the 95% CI. *N/A* not applicable

To demonstrate *epiTEome*’s ability to capture and report the DNA methylation state of TE insertion sites, we used the Arabidopsis Ha-0 ecotype and calculated the average DNA methylation at: (1) each non-reference TE insertion; (2) the flanking non-TE DNA of the insertion site; (3) these same loci without the insertion in the reference Col-0; and (4) the parental TEs that produced the transposed copies in Ha-0 (Fig. 5). We confirmed that transposition of a TE recruits DNA methylation to the insertion site (Fig. 5, middle), which without TE insertion have low DNA methylation levels (Fig. 5, top) [12, 22]. These non-reference TEs and flanking DNA have similar DNA methylation levels compared with their parental TE loci (Fig. 5, bottom). Of note, we observed higher CHH methylation levels at the more recently inserted TEs and flanking sites compared to the parental TE copies, suggesting that the more recent copies are more efficient targets of small RNA-directed DNA methylation. Using a MethylC-seq read of 85 nt, we were able to determine DNA methylation states of 60 bp on either side of the TE insertion site (Fig. 5, right side). This window of resolution will increase with longer sequencing reads; however, it is long enough to detect a difference between DNA methylation contexts at the TE insertion sites: while CG and CHG methylation linearly decrease from the flanks of a more recent TE insertion site, CHH methylation exponentially decreases to a low level within 40 bases of the insertion site (Fig. 5, middle right).

## Discussion

*EpiTEome* represents an improvement both in the sensitivity of TE insertion site detection (Fig. 2a), as well as the ability to use MethylC-seq data. However, there are technical and biological limitations that must be imposed on *epiTEome* to reduce the number of FPs. For example, sequencing depth greatly impacts *epiTEome* sensitivity (Fig. 2c). Increased sequencing depth increases the likelihood of reads with discordant breakpoint junctions near their center. Due to the reduced complexity of the DNA code using bisulfite-converted DNA, the number of non-bisulfite-induced SNPs between the reads and reference genome also significantly alters *epiTEome* sensitivity (Fig. 2c). This will limit TE insertion site detection in lines without a closely related reference genome and will work better for investigation of tissues or mutants within one strain, rather than between multiple strains.

In addition to mapping limitations, *epiTEome* filtering steps remove TE insertion sites within a TE of the same family. This limits insertion detection for TEs that both compose and target heterochromatin, such as some LTR retrotransposons, which are often inserted into similar TE copies [23]. This filtration was necessary to avoid the split-read detection of frequent TE internal deletions produced by non-homologous recombination (not real transposition events). *EpiTEome* will also be more sensitive to detect germinal transposition events rather than somatic. Germinal events will be present in all of the sampled DNA and thus appear in multiple reads along with a correlation of decreased un-split read coverage at that TE insertion site. Somatic insertion split-reads may be rare (depending on the timing/size of the sector) and likely will not have an observable decrease of un-split read coverage.

The major breakthrough of *epiTEome* is the ability to detect new TE insertion sites from a data source designed to detect cytosine DNA methylation. *EpiTEome* is efficient at TE insertion site detection, but also will identify the DNA methylation status of those sites. Even with both genome resequencing and MethylC-seq data in hand, the methylation of new TE insertion sites has not been investigated because both of these datasets are mapped to the reference genome, failing to identify non-reference TE positions. Our proof-of-principle results on one Arabidopsis mutant, one Arabidopsis ecotype, and between two maize inbred lines demonstrates that *epiTEome* can assay DNA methylation at new TE insertion sites in both simple and complex/repetitive genomes. We confirm DNA methylation recruitment to new TE insertion sites and a differential level of methylation between the non-reference and parental TE insertions. By generating the *epiTEome* program, we have provided the epigenetics community the resource to obtain more out of their data and assay two new features:TE insertion sites and their DNA methylation status, from existing or future MethylC-seq data.

## Conclusions

*EpiTEome* provides, for the first time, the ability to detect new TE insertion events from new or existing MethylC-seq data. This program combines the analysis of new TE insertion sites with the analysis of TE DNA methylation into a single analysis, reducing the requirement to perform both genome resequencing and MethylC-seq on the same samples. *EpiTEome* enables new epigenomic investigation of previously overlooked non-reference TE insertion sites.

## Methods

### Identification of new TE insertion sites

All FASTQ files were trimmed for adapters and preprocessed to remove low quality reads using *cutadapt* [24] with the following parameters: -q 30 –max-n 0. Trimmed FASTQ reads were then mapped to a reference genome using the MethylC-Seq mapping program *Bismark* [14] using the following parameters: --bowtie2 --ambiguous --unmapped –R 10 –score_min L,0,-0.6 -N 1. Identification of new TE insertion sites was performed using *epiTEome* (Fig. 1a). As output, *epiTEome* provides a file that contains the coordinates of the non-reference TE insertion sites, the type of mobile TEs, and the parental TE copy. In addition, *epiTEome* also outputs the methylation level at the edge of the non-reference TE and flanking insertion site.

### Simulated data

For the analysis in Fig. 2a and b, 14 different types of TEs were inserted into a total of 84 loci on Arabidopsis chromosome 2. Three different TE insertion contexts (genic, intergenic, and TE) were chosen for each of the 84 neo-insertion sites, generating a synthetic chromosome 2 FASTA sequence. Among the three different TE insertion contexts, TE insertion sites were randomly chosen using *bedtools shuffle* and TEs were inserted using the custom Perl script *insertTEsintoFasta.pl* (available as part of *epiTEome* software package). We next *in silico* bisulfite-converted DNA sequencing reads with *Sherman* [25], using the rates of 20% CG and 3% CH, which represent the average methylation level on Arabidopsis chromosome 2. *In silico* sequencing reads were produced using *Sherman* with a read length of 85 nt, a 20x genome coverage, and no SNPs. All three programs were launched on either the non-bisulfite-converted reads (TEPID and READSPLITER) or bisulfite-converted reads (*epiTEome*) and the outputted coordinates of TE neo-insertion sites were compared with the reference using *bedmap* [26]. This comparison allowed us to estimate the accuracy of each program by calculating the sensitivity (TP/(TP + FN)) and the FDR (FP/(TP + FP)). The same procedure was reproduced for the analysis in Fig. 2c using only *epiTEome*, 84 random insertion sites, and by altering independently the variables: depth of coverage; read length; number of SNPs; and percentage of methylation. A similar procedure was performed for Fig. 4a–c: LTR retrotransposons and DNA transposons were selected from TE families having a low (1–10), medium (11–100), and high (>101) copy number of TEs. Four hundred TEs were randomly inserted within three different TE insertion contexts (genic, intergenic, and TE) of maize chromosome 1 or rice chromosome 1.

### *EpiTEome* analysis of the Ha-0 accession

Coordinates of new TE insertion sites from the Arabidopsis ecotype Ha-0 identified by either SPLITREADER and TEPID were downloaded from [12] and [13]. Comparison of the new TE insertion site coordinates predicted by *epiTEome* with the two other methods was performed using *bedmap*. The proportional Venn diagram in Fig. 3a was produced using *eulerAPE* [27]. Validation of new TE insertion sites detected specifically by *epiTEome* was performed by calculating the un-split read coverage at each new TE insertion site (+/− 80 bp). In Fig. 3e, the un-split read coverage standard score at the insertion site is clustered using the Ward’s method of hierarchical cluster analysis (R package *ggdendro*).

### Analysis of non-reference TE insertions in maize

The coordinates of new TE insertion sites were identified by *epiTEome* by aligning 100 bp bisulfite-converted reads to the maize reference genome (version 3) and corresponding version 3 annotation [28]. *EpiTEome* displayed enhanced sensitivity using the improved version 4 B73 reference sequence and annotation (unpublished data).

### Analysis of DNA methylation level

DNA methylation level was assayed in each of the three cytosine contexts (CG, CHG, and CHH) at the flanking DNA surrounding the new TE insertion site and at both edges of the newly inserted TE. Only MethylC-seq discordant split-reads identified by *epiTEome* were used to calculate this methylation level. Meta-analysis was performed by averaging methylation levels at the 5′ and 3′ of the new insertion site within 10 nt windows. For any given window, the variation in methylation across all elements was used to calculate the 95% CI.

## Availablility and Requirements

*EpiTEome* is freely available at https://github.com/jdaron/epiTEome under a GNU General Public License and the source code has been deposited at Zenodo (doi: 10.5281/zenodo.495189). The general procedure by which *epiTEome* functions is described in Fig. 1 and in more detail within the README file distributed with the software package. *EpiTEome* requires the following list of dependencies: Perl, BioPerl, samtools,bedtools, ngsutils, and segemehl.
